# Wheat *NAM* genes regulate the majority of early monocarpic senescence transcriptional changes including nitrogen remobilization genes

**DOI:** 10.1093/g3journal/jkac275

**Published:** 2022-10-13

**Authors:** Tayyaba Andleeb, Emilie Knight, Philippa Borrill

**Affiliations:** Department of Crop Genetics, John Innes Centre, Norwich Research Park, Norwich NR4 7UH, UK; Department of Plant Sciences, Faculty of Biological Sciences, Quaid-i-Azam University, Islamabad 15320, Pakistan; Department of Crop Genetics, John Innes Centre, Norwich Research Park, Norwich NR4 7UH, UK; Department of Crop Genetics, John Innes Centre, Norwich Research Park, Norwich NR4 7UH, UK

**Keywords:** *Triticum aestivum* L. (wheat), senescence, transcription factors, nitrogen remobilization, flag leaf, *NAM-B1*, *Gpc-B1*, Plant Genetics and Genomics

## Abstract

Senescence enables the remobilization of nitrogen and micronutrients from vegetative tissues of wheat (*Triticum aestivum* L.) into the grain. Understanding the molecular players in this process will enable the breeding of wheat lines with tailored grain nutrient content. The *NAC* transcription factor *NAM-B1* is associated with earlier senescence and higher levels of grain protein, iron, and zinc contents due to increased nutrient remobilization. To investigate how related *NAM* genes control nitrogen remobilization at the molecular level, we carried out a comparative transcriptomic study using flag leaves at 7 time points (3, 7, 10, 13, 15, 19, and 26 days after anthesis) in wild type and *NAM* RNA interference lines with reduced *NAM* gene expression. Approximately 2.5 times more genes were differentially expressed in wild type than *NAM* RNA interference plants during this early senescence time course (6,508 vs 2,605 genes). In both genotypes, differentially expressed genes were enriched for gene ontology terms related to photosynthesis, hormones, amino acid transport, and nitrogen metabolism. However, nitrogen metabolism genes including *glutamine synthetase* (*GS1* and *GS2*), *glutamate decarboxylase* (*GAD*), *glutamate dehydrogenase* (*GDH*), and *asparagine synthetase* (*ASN1*) showed stronger or earlier differential expression in wild-type than in *NAM* RNA interference plants, consistent with higher nitrogen remobilization. The use of time course data identified the dynamics of *NAM*-regulated and *NAM*-independent gene expression changes during senescence and provides an entry point to functionally characterize the pathways regulating senescence and nutrient remobilization in wheat.

## Introduction

Wheat supplies approximately 20% of calories in the human diet and is an important source of protein and micronutrients. Beyond nutritional benefits, wheat grains with higher protein content are associated with increased breadmaking quality and attract a price premium. Although nitrogen (N) fertilization is commonly used to increase grain protein content, high nitrogen fertilization leads to higher production costs and environmental pollution (Aranguren *et al.* 2021; Martínez-Dalmau *et al.* 2021). Alternatively, genetic approaches can be used to increase protein content, although identifying the genetic loci to target remains a challenge.

The final grain yield and nutrient content depends on the accumulation and transport of carbon, nitrogen and other nutrients from the vegetative tissues to the developing grain. The remobilization of nutrients is strongly influenced by the process of senescence, which is a developmentally regulated programme to remobilize nutrients from vegetative tissues to the developing grain. The starting time and progression of flag leaf senescence influences the remobilization of nutrients and the final yield (Distelfeld *et al.* 2014), with the flag leaf contributing a significant proportion of nitrogen to the seed by degrading and recycling proteins (Kichey *et al.* 2007; Bogard *et al.* 2010; Havé *et al.* 2017). Delayed leaf senescence can be associated with prolonged photosynthesis and increased grain yield but also decrease grain protein content due to reduced nutrient remobilization from the leaf tissues (Uauy *et al.* 2006; Alpuerto *et al.* 2021). Therefore, altering the rate and progress of senescence can influence final yield and protein content of wheat grain. Understanding the molecular components influencing flag leaf senescence and nitrogen remobilization can help to improve nitrogen remobilization efficiency and grain protein content in wheat.

The identification of the *NAM-B1* gene which is a NAC transcription factor that influences senescence and grain nutrient content opens the door to identify the molecular pathways regulating senescence and nutrient remobilization in wheat. *NAM-B1* was identified through positional cloning as the causal gene for *Gpc-B1* which affects grain protein content (Uauy *et al.* 2006). *NAM-B1*, together with its homoeologs *NAM-A1* and *NAM-D1*, influences senescence and enhances nutrient remobilization (Avni *et al.* 2014; Cormier *et al.* 2015; Harrington *et al.* 2019). Most modern wheat cultivars carry a nonfunctional allele of *NAM-B1*, whereas the functional allele, which was identified through map-based cloning, is mainly found in wild emmer wheat and landraces (Hagenblad *et al.* 2012). Closely related paralogs of *NAM-B1* have been identified on chromosome 2 which also regulate senescence and nutrient remobilization (*NAM-A2*, *NAM-B2*, and *NAM-D2*) (Pearce *et al.* 2014; Borrill *et al.* 2019). A study of *NAM* RNAi lines with reduced expression of the *NAM-B1* homoeologs and paralogs showed that remobilization of micronutrients and nitrogen was strongly reduced in the *NAM* RNAi lines, which directly implicates *NAM* genes in the control of nutrient remobilization during senescence (Waters *et al.* 2009). These *NAM* genes provide a valuable entry point to decipher the control of monocarpic senescence and nitrogen remobilization in wheat at the molecular level.

A transcriptomic study of the same *NAM* RNAi lines at 12 days after anthesis revealed that *NAM* genes regulate transporters, hormone-regulated genes and transcription factors at this early stage of senescence in flag leaves (Cantu *et al.* 2011). Additional *NAM*-regulated genes in flag leaves were identified by comparing wild-type (WT) plants to lines mutated in either *NAM-A1* or *NAM-A1* and *NAM-B2* at 0, 12, and 22 days after anthesis using a tetraploid genotype carrying a *NAM-B1* deletion (Pearce *et al.* 2014). Consistent with Cantu *et al.* (2011), *NAM*-regulated genes included photosynthesis genes and many zinc and iron transport genes. These studies provide a valuable insight into the transcriptional effects of *NAM* genes, but the small number of timepoints limits our ability to understand the influence of *NAM* genes throughout monocarpic senescence. Furthermore, reduced sequencing costs and advances in genome assemblies and annotation for wheat allow more accurate analysis than was possible when previous studies on *NAM*-regulated genes were carried out using de novo transcriptome assemblies (Cantu *et al.* 2011) or earlier genome assemblies (Pearce *et al.* 2014; Harrington, Backhaus, Singh, *et al.* 2020).

Studies using time course data can reveal the dynamics of gene expression during a developmental process. Previous studies have characterized changes in flag leaves at the transcriptome level during senescence in wheat (Zhang *et al.* 2018; Borrill *et al.* 2019), but we do not have a full understanding of the timing of gene expression controlled by *NAM* genes for nutrient remobilization during monocarpic senescence.

To address the lack of time-resolved understanding of *NAM* gene regulation of senescence and nutrient remobilization, we analyzed flag leaf tissues at 7 time points from WT and *NAM* RNAi wheat plants. Previous work demonstrated that *NAM* genes strongly influence nitrogen remobilization but the downstream molecular pathways were largely unknown. Therefore, we characterized gene expression changes in nitrogen-associated genes during senescence in WT and *NAM* RNAi plants and identified genes through which *NAM* genes may influence nitrogen remobilization. These putative *NAM* gene targets may represent target genes to improve nitrogen remobilization in wheat.

## Methods

### Plant material and growth condition

WT wheat (*Triticum aestivum*) plants cv. Bobwhite and sibling lines with reduced levels of *NAM* gene expression (*NAM* RNAi) were generated by Uauy *et al.* (2006). All plants were grown as previous described in Borrill *et al.* (2019) and the samples analyzed in this article for the WT are a subset of those previously published in Borrill *et al.* (2019). WT and *NAM* RNAi plants were grown together in the same growth room.

Briefly, we pregerminated WT and *NAM* RNAi seeds on Whatman filter paper for 48 h at 4°C, followed by 48 h at ∼20°C. These germinated seeds were then sown in trays (P40) containing a mixture of horticultural grit (15%) and fine peat (85%). We transferred individual plants to 1-l square pots containing Petersfield Cereal Mix at 2–3 leaf stage. Plants were grown in light (16 h) and dark (8 h) at the temperature of 20 and 15°C, respectively. We tagged the main tiller in each pot for anthesis date, phenotyping, and sample collection.

### Phenotypic data collection

We used SPAD-502 chlorophyll meter (Konica Minolta) to measure the flag leaf chlorophyll content at 7 time points [3, 7, 10, 13, 15, 19, and 26 days after anthesis (DAA)]. At each time point, we recorded chlorophyll content from 5 independent plants, measuring 8 locations along each flag leaf length, using only the tagged main tiller. Three out of 5 leaves measured for chlorophyll content were subsequently harvested for RNA extraction.

We measured grain moisture content at the same 7 time points (3, 7, 10, 13, 15, 19, and 26 DAA) at which we measured leaf chlorophyll content. From 5 independent plants, we harvested 8 grains from the central spikelets (floret positions 1 and 2) from the tagged spike at each time point. We weighed fresh grains, then reweighed them after drying at 65°C for 72 h to obtain dry weight. We calculated the % grain moisture content from the difference in fresh and dry weight of a seed.

### Sample collection

For RNA extraction, we harvested the flag leaf from the tagged main tiller at 7 time points: 3, 7, 10, 13, 15, 19, and 26 DAA for both WT and RNAi lines. From each flag leaf we harvested the middle 3 cm lengthways, to focus on a developmentally sychronized section of tissue. One flag leaf section was harvested for each of the 3 independent replicates for each timepoint and genotype. The samples were flash frozen in liquid nitrogen and stored at −80°C.

### RNA extraction

We ground the leaf samples to a fine powder using mortar and pestles prechilled with liquid nitrogen. RNA was extracted using TRIzol by following the manufacturer’s (ThermoFisher) protocol, with 1 ml TRIzol added to 100 mg ground samples. Genomic DNA contamination was removed by using DNAsel (Qiagen) and samples were further cleaned through RNeasy Minikit by following instructions of the manufacturer (Qiagen).

### qRT-PCR

RNA samples from WT and RNAi lines sampled at 19 DAA were also used in a qRT-PCR assay. They were normalized to 250 ng/µl prior to DNAseI treatment (Thermo Scientific) at 37°C for 30 min. The reaction was terminated by the addition of DNase-Stop solution and incubation at 65°C for 10 min to inactivate the DNase.

Reverse transcription was performed with the Invitrogen M-MLV Reverse Transcriptase (Invitrogen). For each sample, annealing was carried out by incubating 11 µl of DNase-treated RNA + 1 µl dNTPs (10 mM) + 1µl oligo (dT) 12–18 Primer (diluted 1:2) (Invitrogen) + 1 µl random primers (diluted 1:20) (Invitrogen) at 65°C for 5 min. cDNA synthesis was performed in a thermal cycler, by the addition of 1 µl M-MLV reverse transcriptase, 4 µl of 5× first strand buffer, 2 µl of 0.1 M DTT, and 1 µl of Ribolock RNase Inhibitor (Thermo Scientific) to the 14 µl of annealed sample. The program consisted of a 25°C incubation for 10 min, followed by a 37°C incubation for 50 min and a final 70°C incubation for 15 min to inactivate the reverse transcriptase.

cDNA samples were used in a qRT-PCR assay using primers featured in Supplementary Table 9 of Uauy *et al.* (2006). qRT-PCR assay was carried out using LightCycler 480 SYBR Green I Master Mix on a LightCycler 480 instrument as described in Harrington, Backhaus, Fox, *et al.* (2020), following the same conditions.

Three technical replicates were carried out per sample. Transcript levels (Ct values) were normalized using the ΔCt method, using the *ACTIN* values as the internal control. The ΔΔCt values were further calculated using the average ΔCt of the 3 WT values. 2^−ΔΔCT^ values for RNAi lines were plotted relative to the expression of the WT (% expression where WT is 100%).

### Library preparation and sequencing

Library preparation and sequencing were carried out using the same methods as described in Borrill *et al.* (2019). Briefly after RNA quality confirmation, the TruSeq RNA protocol v2 was used for the construction of TruSeq RNA libraries on PerkinElmer Sciclone (Illumina 15026495 Rev.F). After adaptor ligation, the libraries were size selected using Beckman Coulter XP beads (A63880). The PCR used a primer cocktail, which enriched DNA fragments having adaptors at both ends. Library insert sizes was confirmed by running an aliquot of the DNA library on a PerkinElmer GX (PerkinElmer CLS760672) and concentration measured using the Tecan plate reader.

After normalization, the TruSeq RNA libraries were equimolar pooled into 2 final pools using Qiagen elution buffer (1 pool contained WT samples, 1 pool contained RNAi samples). Each library pool was diluted to a 2 nM concentration using 0.2 N sodium hydroxide (NaOH). Five microliters of this solution was added to 995 μl of HT1 (Illumina) to give a final concentration of 10 pM. The diluted library pool (120 μl) was spiked with PhiX control v3 (1% v/v) and transferred to a 200-μl strip tube and placed on ice before loading on the Illumina cBot. The HiSeq PE Cluster Kit v3 was used to cluster the flow cell on the Illumina cBot, using the Illumina PE_Amp_Lin_Block_Hyb_V8.0 protocol. After clustering the flow cell was transferred onto the Illumina HiSeq 2000/2500 instrument. The sequencing chemistry was HiSeq SBS Kit v3 coupled with HiSeq Control Software 2.2.58 and RTA 1.18.64. Reads in bcl format were demultiplexed using the 6-bp Illumina index by CASAVA 1.8, allowing for a 1 base-pair mismatch per library, and converted to FASTQ format by bcl2fastq.

### Transcriptome analysis—mapping

We pseudoaligned the samples using Kallisto v0.44.0 with default settings to the RefSeqv1.0 annotation v1.1 (International Wheat Genome Sequencing Consortium (IWGSC) 2018). We noticed that unexpectedly the *NAM* genes were more highly expressed in the *NAM* RNAi lines than the WT lines. Examining the read alignment we found that the transgenic RNAi construct was mapping to the *NAM* gene transcripts and artificially inflating *NAM* gene expression levels in these samples. To account for this, we substituted these regions of anomalous mapping in each of the *NAM* gene transcripts with Ns (613–623 bp, representing on average 29.3% of the transcript length; *TraesCS2A02G201800.1*, *TraesCS2A02G201800.2*, *TraesCS2B02G228900.1*, *TraesCS2B02G228900.2*, *TraesCS2D02G214100.1*, *TraesCS6A02G108300.1*, *TraesCS6A02G108300.2*, *TraesCS6D02G096300.1*, *TraesCS6B02G207500LC.1*, *TraesCS6B02G207500LC.2*). Samples were re-mapped to this masked version of the v1.1 annotation and all subsequent analysis used these re-mapped values. Reads were still able to map to the remaining portions of the *NAM* gene transcripts (∼70% of each transcript length). The masked v1.1 annotation is available at https://doi.org/10.6084/m9.figshare.20210774.v1. In total, we analyzed 42 samples: 3 replicates of 7 timepoints (3, 7, 10, 13, 15, 19, and 26 DAA) for 2 genotypes (WT and *NAM* RNAi). For comparison, the count and TPM (transcripts per million) of all samples were combined into 1 data frame by using tximport v1.0.3 (Soneson *et al.* 2015). All scripts used for the data analyses in this article are available at https://github.com/Borrill-Lab/NAM_RNAi_Senescence and input files required to run the scripts can be found at https://doi.org/10.6084/m9.figshare.20210774.v1.

### Differential expression analysis

We filtered the data for further analysis to include only high confidence genes; expressed at >0.5 TPM at least in 1 time point. This strategy excluded all low confidence gene models and low expressed genes from the data (Ramírez-González *et al.* 2018). In total, 52,395 genes in WT and 52,626 genes in RNAi were expressed at >0.5 TPM. We identified genes that were differentially expressed at each timepoint by comparing WT and RNAi samples using DESeq2 v1.14.1 (Love *et al.* 2014). We then analyzed the data using time-aware differential expression analysis software. The count expression levels of the genes expressed >0.5 TPM were rounded to the nearest integer to identify differentially expressed genes (DEGs) using ImpulseDE2 v1.10.0 (Fischer *et al.* 2018). For accuracy, we also identified DEGs through Gradient Tool v1.0 (Breeze *et al.* 2011) by using the TPM expression level of 52,395 genes in WT and 52,626 genes in RNAi on Cyverse (https://de.cyverse.org/de/) with enabled data normalization option (Merchant *et al.* 2016). To identify high confidence gene DEGs, we filtered to only consider genes as differentially expressed that were both identified by using ImpulseDE2 at *P*_adj_ < 0.001 and Gradient Tool at *z*-score of >|2|.

### Group patterns of differentially expressed genes

We categorized the high confidence DEGs on basis of the first-time point at which they were either up- or downregulated according to Gradient Tool output for the WT and RNAi time courses separately. The Gradient Tool is based on Gaussian process regression for the identification of gene expression patterns either increasing (upregulated) or decreasing (downregulated) at each time point (Breeze *et al.* 2011). A gene that was first upregulated at 7 DAA was placed in the “U07” group (up 7 DAA). While a gene that was first downregulated at 7 DAA was categorized in the “D07” group (down 7 DAA). Few genes (∼2% of all DEGs) were both up- and downregulated during either time course (3–26 DAA); these were assigned a group based on their first expression pattern with the opposite trend also indicated. For instance, a gene that showed downregulation at 7 DAA and then upregulated at 19 DAA was grouped as “D07U” (the second time point at which differential expression occurred was not reported in the grouping pattern). These grouping patterns for WT and RNAi are available in Supplementary Tables 1 and 2, respectively. The genes with both up- and downregulation trends (∼2% of all DEGs) were excluded from further analyses.

### Gene ontology term enrichment

Gene ontology (GO) terms were only available for the RefSeqv1.0 annotation; therefore, we used the same approach as Borrill *et al.* (2019) to transfer GO terms to the v1.1 annotation. We only transferred GO terms for genes which were >99% identical across >90% of the sequence (105,182 genes; 97.5% of all HC genes annotated in v1.1). Using GOseq v1.38.0 (Young *et al.* 2010), GO term enrichment was done for each group of DEGs separately [groups were assigned based on the first time point gene expression pattern either increasing (upregulated) or decreasing (downregulated)].

### Nitrogen orthologs identification

We identified a list of genes involved in nitrogen metabolism in *Arabidopsis* through a literature search (Su *et al.* 2004; Grallath *et al.* 2005; Hirner *et al.* 2006; Stacey *et al.* 2006; Masclaux‐Daubresse *et al.* 2008; Havé *et al.* 2017; Gaudinier *et al.* 2018; Brumbarova and Ivanov 2019). We then identified their respective orthologs in wheat using EnsemblPlants ortholog information downloaded via BioMart (Kersey *et al.* 2018). Due to the evolutionary distance between *Arabidopsis* and wheat it was not possible to assign 1:1 orthologs in many cases due to within-lineage duplications and gene losses. Therefore, we took an inclusive approach to identifying orthologs, considering that all wheat genes in the gene tree could be orthologs of the associated *Arabidopsis* gene (Supplementary Table 3). Functional annotation of nitrogen-associated genes differentially expressed in WT and RNAi was obtained from literature searches and g:Profiler (Raudvere *et al.* 2019).

## Results

### Phenotypic data and *NAM* gene expression

**Fig. 1.**
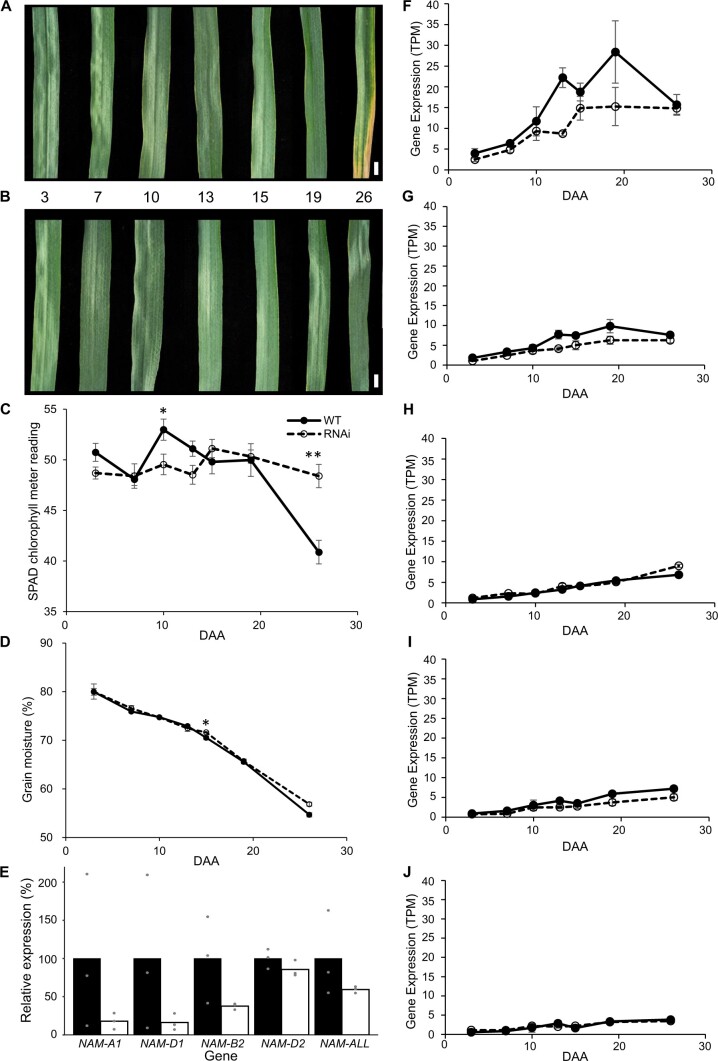
Characterization of WT and *NAM* RNAi plants in time course of flag leaf senescence from 3 to 26 DAA. a and b) Flag leaf images of WT and *NAM* RNAi from 3 to 26 DAA [WT images in (a) originally published in Borrill *et al.* 2019]. c) SPAD chlorophyll meter readings for flag leaves across the time course from 3 to 26 DAA, *n* = 5. d) Grain moisture content of grains across the time course from 3 to 26 DAA, *n* = 5. e) Relative expression levels of *NAM* genes measured by qRT-PCR in WT (black) and RNAi plants (white), *n* = 3, with individual datapoints shown as gray dots. Expression is relative to the average of the 3 WT samples (100%). f–j) Expression pattern of *NAM-1* and *NAM-2* genes 3–26 DAA in WT and *NAM* RNAi measured using RNA-Seq. f) *NAM-A1* (*TraesCS6A02G108300*), g) *NAM-D1* (*TraesCS6D02G096300*), h) *NAM-A2* (*TraesCS2A02G201800*), i) *NAM-B2* (*TraesCS2B02G228900*), and j) *NAM-D2* (*TraesCS2D02G214100*). Error bars represent standard error of the mean. *n* = 5 for SPAD chlorophyll meter reading and grain moisture content and *n* = 3 for gene expression data. Scale bar = 1 cm.

To examine the transcriptional differences during the initiation of senescence in WT and plants with reduced *NAM* gene expression (*NAM* RNAi), we harvested an early time course of flag leaf senescence at 3, 7, 10, 13, 15, 19, and 26 DAA (Fig. 1, a and b). SPAD chlorophyll meter readings recorded from the flag leaves were similar from 3 to 19 DAA in both WT and RNAi, with a significantly reduced value at 26 DAA in WT compared to RNAi (Fig. 1c). Grain moisture content decreased significantly between 3 and 26 DAA for both genotypes at a similar rate. By 26 DAA, the grain moisture content (55% in WT and 57% in RNAi) indicated that the wheat plants had reached soft dough stage (GS85) and the time period sampled included the majority of the grain-filling period (Fig. 1d; Zadoks *et al.* 1974). To further validate these materials we measured *NAM* gene expression using previously reported qRT-PCR primers (Uauy *et al.* 2006) and found that *NAM* gene expression was lower on average in RNAi compared to WT flag leaves at 19 DAA (Fig. 1e). Expression of the RNAi construct was only detected in the RNAi samples (Supplementary Table 4). We found that as expected, *NAM-A1* and *NAM-D1* were expressed at lower levels in the *NAM* RNA interference (RNAi) line compared to WT at same 7 time points for which phenotypic data were recorded (Fig. 1, f and g). The expression pattern of *NAM-B1* is not reported as this homoeolog is deleted in cv. “Bobwhite” used in this study (Uauy *et al.* 2006). The *NAM2* homoeologs were expressed at lower levels than *NAM1* (Fig. 1, f–j) with smaller differences between WT and RNAi.

### Transcriptome profile in WT and RNAi during senescence

#### WT plants had stronger transcriptional changes than RNAi during the time course

RNA was extracted from the flag leaf and sequenced for each of the 7 time points. RNA-seq data were aligned using kallisto (Bray *et al.* 2016) to the RefSeqv1.1 transcriptome annotation (International Wheat Genome Sequencing Consortium (IWGSC) 2018). Initially we observed artificially high levels of *NAM* gene expression in the *NAM* RNAi samples. Examining the read alignments this was caused by mapping of transcripts from the transgenic *NAM* RNAi construct to the *NAM* genes. Therefore we masked these regions of the coding sequence of the *NAM* genes with Ns to prevented artificial inflation of *NAM* gene expression in the *NAM* RNAi samples (on average 29% of the *NAM* coding sequence was masked). After remapping to the RefSeqv1.1 transcriptome with masked regions in the *NAM* genes, on average samples had 33.7M reads and 27.5M reads were pseudoaligned by kallisto (81.3%) (Supplementary Table 5). *NAM* gene expression was still detectable using the remaining ∼70% of each *NAM* transcript (Fig. 1, f–j).

As a first step to understanding transcriptional differences between WT and RNAi we compared gene expression at each time point individually. In most timepoints <80 genes were upregulated in WT compared to RNAi, except at 26 DAA when 549 genes were upregulated (>2-fold change, FDR <0.001; Supplementary Tables 6 and 7). The 549 genes upregulated in WT at 26 DAA were enriched for GO terms associated with senescence and chlorophyll catabolism (*P*_adj_ < 0.05; Supplementary Table 7). More genes were downregulated than upregulated at every time point, with a range from 99 to 874 downregulated genes. The largest number of downregulated genes occurred at the start and end of the time course. At the earliest timepoint 3 DAA, 693 genes were downregulated in WT compared to RNAi (>2-fold change, FDR <0.001; Supplementary Table 7) and these were enriched for GO terms associated with catabolic processes and response to freezing. At the final timepoint 874 genes were downregulated in WT compared to RNAi and these were enriched for GO terms related to photosynthesis. None of the *NAM* genes (Fig. 1) were identified as differentially expressed between WT and RNAi by DESeq2, which may be due to variability between replicates and stringent *P*-value and fold change thresholds. Although this pairwise analysis identifies genes differentially expressed at each timepoint, it ignores information from adjacent timepoints and does not provide information on individual gene expression trajectories over the time course. Therefore, we decided to identify DEGs in each genotype separately over time to reveal how dynamic gene expression is affected by the reduction in *NAM* gene expression in the RNAi lines compared to WT. We hypothesized that this approach would identify how the knock-down of *NAM* genes affects the overall senescence transcriptional programme and provide time-specific information.

**Fig. 2.**
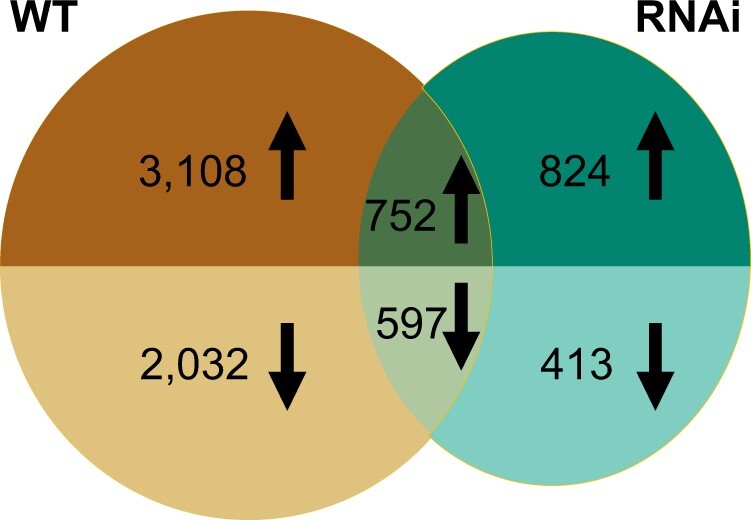
Venn diagrams of DEGs in WT and *NAM* RNAi across time. ImpulseDE2 and Gradient Tool were used to identify the DEGs during the timecourse (3–26 DAA) in WT and RNAi separately. Upregulated genes are shown in the top half of each circle and downregulated genes in the bottom half of each circle. The intersection of 2 circles represents genes differentially expressed in both WT and RNAi. Out of 1,368 common DEGs, 19 genes were upregulated in 1 genotype and downregulated in the other (not shown).

To identify differently expressed genes in both WT and RNAi, we used ImpulseDE2 and Gradient Tool. We found that from 3 to 26 DAA 6,508 (WT) and 2,605 (RNAi) genes were differentially expressed. In WT, out of 6,508 DEGs, 3,870 genes were upregulated and 2,638 genes were downregulated (Fig. 2 and Supplementary Table 1). While in RNAi, out of 2,605 DEGs, 1,585 genes were upregulated and 1,020 genes were downregulated (Fig. 2 and Supplementary Table 2). During the time course, more genes were upregulated than downregulated in both WT and RNAi. This suggests that senescence is actively controlled through transcriptional upregulation rather than general downregulation in wheat. Approximately half of the DEGs in RNAi were also found in WT (Fig. 2); contrastingly, most DEGs in WT were not differentially expressed in RNAi, suggesting a unique transcriptional response in WT compared to RNAi.

#### An initial wave of downregulation is followed by upregulation of gene expression in both genotypes

To understand the temporal nature of gene expression changes, we assigned DEGs (6,508 in WT and 2,605 in RNAi) into groups according to the first time they were up- or downregulated. For instance, a gene first upregulated at 7 DAA would be grouped as “U07” (up 07 DAA), and a gene that first showed downregulation at this time point would be grouped as “D07.” We found that less than 2% of genes were up- and then downregulated or vice versa during the time course in either WT (1.4%) or RNAi (1.8%) and these were excluded from further analysis. The remaining 98% of genes were described by 13 expression patterns in WT and RNAi (Supplementary Table 8).

**Fig. 3.**
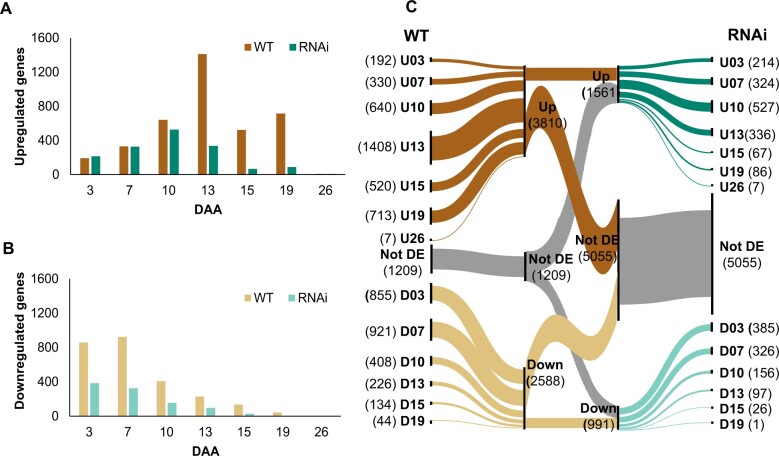
Differential expression of genes across the time course in WT and *NAM* RNAi plants. Genes are grouped according to the first time they were up- or downregulated. a) Upregulated genes, b) downregulated genes, and c) alluvial plot showing comparison of differential expression patterns in WT and RNAi. In (c), the number in brackets for each group pattern represents number of DEGs at that time point. Not DE stands for not differentially expressed.

In WT and RNAi, up- and downregulation patterns were not evenly spaced over time. In both WT and RNAi, the number of genes starting to be upregulated increased during the early time points from 3 to 10 DAA, but from 13 DAA onwards the number of genes upregulated in RNAi fell to a lower level, whereas in WT 13 DAA was the timepoint with the highest number of genes upregulated (Fig. 3a). More genes were upregulated in WT than RNAi at later stages of senescence (13 to 26 DAA). With the onset of chlorophyll loss at the end of the time course (26 DAA; Fig. 1a), very few genes showed differential expression in either WT or *NAM* RNAi (7 genes upregulated in each line). Initiation of downregulation was stronger in the early stages of the time course in both lines, with more genes downregulated in WT than RNAi (Fig. 3b). As senescence progressed, only a limited number of genes were downregulated; 44 genes at 19 DAA in WT. In both WT and RNAi no gene was downregulated at 26 DAA suggesting that senescence process is actively regulated through transcriptional upregulation at later stages of senescence (Fig. 3a). A major shift from downregulation at the start of senescence to upregulation enduring the middle and later timepoints is evident in our dataset (Fig. 3, a and b).

We found that the most of DEGs were up- or downregulated at different timepoints in WT and RNAi. For example, in WT at 3 DAA (U03) 192 genes were upregulated but in RNAi only 62 of these genes were upregulated at 3DAA while 130 of them were not differentially expressed (Not DE; Fig. 3c). This limited conservation of expression profiles was common across all timepoints and in both up- and downregulated genes (Fig. 3c). We identified 1,209 genes which were not differentially expressed in WT but showed differential expression in RNAi and an even greater number were differentially expressed in WT but not differentially expressed in RNAi (5,055 genes; Fig. 3c).

#### GO term enrichments in WT and RNAi

**Fig. 4.**
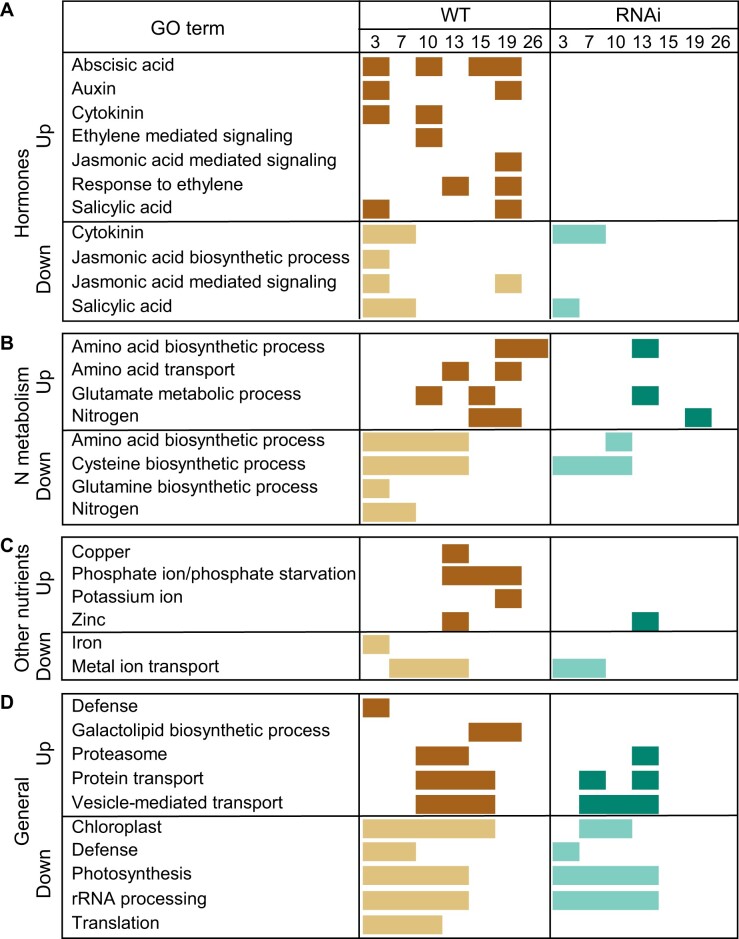
Biological processes enriched in up- and downregulated genes in WT and RNAi lines during a time course (3–26 DAA) of senescence. Filled rectangles indicate that genes starting to be differentially expressed at that time point are enriched for that specific GO term. Enriched GO terms are grouped into a) hormones, b) nitrogen (N) metabolism, c) other nutrients, and d) general processes. Brown rectangles represent upregulated genes in WT; dark green represents upregulated genes in RNAi; pale yellow rectangles represent downregulated genes in WT; and light green rectangles represent downregulated genes in RNAi.

To identify the biological processes and functions associated with each group pattern in our dataset we performed GO enrichment analysis (Fig. 4 and Supplementary Table 8). DEGs in WT were more strongly enriched for GO terms associated with hormones, nitrogen metabolism and other nutrient metabolism than DEGs in RNAi (Fig. 4). Upregulated genes were enriched for hormone signaling and biosynthesis genes in WT but not in RNAi (Fig. 4a). Upregulated genes were enriched for GO terms associated with protein transport, proteasome, vesicle mediated transport and expressed at later time points in WT compared to RNAi (Fig. 4d). Genes enriched for GO terms associated with housekeeping functions such as chloroplasts, photosynthesis, rRNA processing, and translation were downregulated at more timepoints in WT compared to RNAi (Fig. 4d).

The differential expression patterns of genes enriched with N-associated GO terms were more obvious in WT than RNAi. GO terms related to nitrogen (N) metabolism such as nitrogen and amino acid transport, glutamine, glutamate, cysteine biosynthesis were mostly downregulated early in the time course and then upregulated in both WT and RNAi, although the upregulation was less extensive in RNAi (Fig. 4b). Genes enriched with GO terms associated with other nutrients such as copper, phosphate, potassium, and zinc showed upregulation in WT but most of them were not enriched in RNAi except zinc at 13 DAA (Fig. 4c). Genes enriched with GO terms associated with metal ion transport were downregulated at early time points in both WT and RNAi (Fig. 4c). Overall, DEGs in WT had stronger GO term enrichments, with particularly strong enrichment for processes related to hormones and nitrogen metabolism, but these enrichments were less frequently observed in RNAi.

### Genes directly involved in nitrogen metabolism

To identify the effect of *NAM* gene on nitrogen metabolic pathway during time course of senescence, we assembled the list of genes involved in nitrogen metabolism in *Arabidopsis* through literature searches. We then identified their respective orthologs in wheat (*T. aestivum* L.) using *EnsemblPlants* ortholog information downloaded via BioMart. After that, we identified the expression patterns of genes involved in nitrogen transport, assimilation remobilization and transcriptional regulation in WT and RNAi lines. In total we identified 1,027 genes in wheat associated with nitrogen metabolism, of which 587 and 580 genes were expressed during flag leaf senescence in WT and RNAi, respectively. Nitrogen-associated genes were differentially expressed more in WT (136) than RNAi (41) during the time course. The greater number of nitrogen-associated genes DEGs in WT suggests greater changes to nitrogen remobilization or metabolism in WT than RNAi. Overall, nitrogen-associated genes expressed during time course of senescence showed upregulation in WT but most of them were downregulated or not differentially expressed in *NAM* RNAi line indicating that reduced *NAM* genes affects the expression patterns of these genes in wheat.

#### Expression patterns of nitrogen transporters in WT and RNAi

**Fig. 5.**
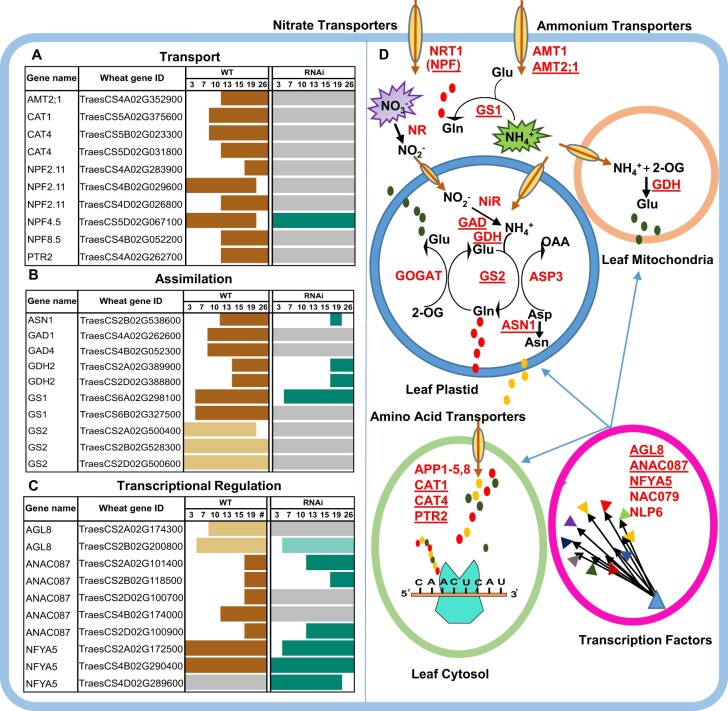
Schematic representation of genes, enzymes, and processes involved in nitrogen metabolism in wheat. The plots present on the left side of figure represent differential expression patterns across time of the 10 most highly expressed genes involved in nitrogen cycling for each category: a) transport, b) assimilation, and c) transcriptional regulation. These genes are red colored, bold, and underlined in the figure to the right (d). Gene names (a–c) are given based on orthology to *Arabidopsis* and orthology is not always 1:1 between *Arabidopsis* and wheat (see Supplementary Table 3). In (a)–(c), brown rectangles represent upregulated genes in WT; dark green represents upregulated genes in RNAi; pale yellow rectangles represent downregulated genes in WT; and light green rectangles represent downregulated genes in RNAi. d) Nitrogen-associated gene pathways in wheat. Ammonium (AMT) and nitrate transporters (NRTs), transport ammonium (${\text{NH}}_{4}^{+}$), and nitrate ions (${\text{NO}}_{3}^{-}$) across the cell membrane. In the cytosol, nitrate reductase (NR) enzyme reduces nitrate to nitrite. Then, nitrite reductase (NiR) reduces nitrite into ammonium in the plastids. After that glutamine synthetase (GS)/glutamine-2-oxoglutarate aminotransferase (GOGAT) cycle assimilates ammonia into N-containing compounds. Asparagine synthetase (ASN) and glutamate dehydrogenase (GDH) are involved in further assimilation of nitrogen compounds into different amino acids. Glu, glutamate; Gln, glutamine; Asn, asparagine; Asp, aspartate; 2-OG, 2-oxoglutarate; OAA, oxaloacetate. These amino acids are then transported to developing grain through different amino acid transporters (AAP, CAT1, CAT4, PTR2). All these steps are regulated by transcription factors (AGL8, ANAC087, NFYA5, NAC079, NLP6).

During senescence, nitrogen is transported via *ammonium* (*AMT2;1*) and *nitrate* (*NRT1.4*, *NFP5.10*, *NRT2.5*) *transporters* across the cell membrane in the form of nitrate (${\text{NO}}_{3}^{-}$) and ammonium (${\text{NH}}_{4}^{+}$) ions (van der Graaff *et al.* 2006; Kong *et al.* 2016). Most nitrate transporters in our dataset were upregulated in WT flag leaves but not differentially expressed in *NAM* RNAi (Supplementary Tables 3 and 9). Similarly, the highly expressed ammonium *transporter* (*AMT2;1*; *TraesCS4A02G352900*) was upregulated in WT but not differentially expressed in RNAi during our time course (Fig. 5a).

The protein degradation occurring in the senescing leaf provides glutamine (Gln), glutamate (Glu) and asparagine (Asn) which are then transported to the seed via amino acid transporters. These amino acid transporters include permeases (*AAPs*), proline transporters (*ProTs*), *ANT1*-like aromatic, and neutral amino acid transporters, γ-aminobutyric acid transporters (GATs) cationic amino acid transporters (*CATs*) and lysine-histidine-like transporters (*LHTs*). The amino acid transporters *CAT1* (*TraesCS5A02G375600*), *CAT4* (*TraesCS5B02G023300*, *TraesCS5D02G031800*), *NPF2.11* (*TraesCS4A02G283900*, *TraesCS4B02G029600*, *TraesCS4D02G026800*), *NPF8.5* (*TraesCS4B02G052200*), and *PTR2* (*TraesCS4A02G262700*) were upregulated in WT but were not differentially expressed in RNAi (Fig. 5a). Interestingly, *NPF4.5* (*TraesCS5D02G067100*) was the only amino acid transporter among 10 highly expressed nitrogen transporters, which was upregulated in both WT and RNAi (Fig. 5a). Many other important nitrogen transporters were also expressed in our data either in WT or RNAi, such as *AAP* (*AAP2*, *AAP3*, *AAP4*, and *AAP8*), *PTR*, *CAT*, *GAT*, *LAT*, *LHT*, *ANT1*, and *NPF*. Most of these amino acid transporters showed upregulation in WT but these were either not differentially expressed or downregulated in RNAi (Fig. 5a and Supplementary Tables 3 and 9).

#### Expression patterns of nitrogen assimilation genes in WT and RNAi

Many genes known to be involved in nitrogen assimilation and remobilization were expressed in our RNA-seq data (Fig. 5b and Supplementary Tables 3 and 10), such as *nitrate reductase* (*NR*), *nitrite reductase* (*NiR*), *glutamine synthetase* (*GS*), *glutamate dehydrogenase* (*GDH*), *glutamate decarboxylase* (*GAD*), and *asparagine synthetase* (*ASN*). In general, nitrogen assimilation and remobilization-related genes were more frequently up- or downregulated in the WT time course than in the RNAi time course (Fig. 5b). Some genes showed later upregulation in RNAi than in WT including *ASN1* (*TraesCS2B02G538600*) and *GDH2* (*TraesCS2A02G389900, TraesCS2D02G388800*). Other genes were upregulated in WT but not differentially expressed in RNAi including *GAD1* (*TraesCS4A02G262600*) and *GAD4* (*TraesCS4B02G052300*). Both *GS1* homoeologs (*TraesCS6A02G298100* and *TraesCS6B02G327500*) were upregulated in WT, but only the A homoeolog was upregulated in RNAi. Three homeologs of *GS2* (*TraesCS2A02G500400*, *TraesCS2B02G528300*, and *TraesCS2D02G500600*) were downregulated in WT but not differentially expressed in RNAi (Fig. 5b).

#### Expression patterns of nitrogen transcriptional regulators in WT and RNAi

In addition to the transporters and enzymes, a number of regulatory TF genes are known in *Arabidopsis* to participate in nitrogen metabolism. In our dataset, the A homoeolog of *AGL8* (*TraesCS2A02G174300*) was downregulated in WT but not differentially expressed in RNAi, while its B homeolog (*TraesCS2B02G200800*) showed downregulation in both WT and RNAi (Fig. 5c and Supplementary Tables 3 and 11). For *ANAC087*, the 5 orthologs were upregulated in WT while 2 of them (*TraesCS4B02G174000* and *TraesCS2D02G100700*) were not differentially expressed in RNAi (Fig. 5c). We also found that genes involved in carbon metabolism which were differentially expressed in WT, were often not differentially expressed in RNAi (Supplementary Table 12). Overall, we found that many more nitrogen-associated genes were up- or downregulated during the senescence time course in WT than in *NAM* RNAi plants (Fig. 5, a–d).

## Discussion

In this study, we compared transcriptional changes in WT and *NAM* RNAi wheat plants associated with flag leaf senescence. We found that approximately 2.5 times more genes were differentially expressed in WT than in RNAi plants from 3 to 26 days after anthesis. Many genes associated with nitrogen metabolism are differentially expressed in WT plants but not in RNAi plants, which is consistent with previously reported phenotypic effects of *NAM* genes on nitrogen remobilization (Uauy *et al.* 2006; Waters *et al.* 2009).

### Dynamic transcriptional changes uncovered through time-aware differential expression analysis

The conventional approach to understand the transcriptional responses to a gene requires pairwise comparison between plants with and without the gene of interest. Using DESeq2, we carried out this pairwise analysis and identified tens to hundreds of genes differentially expressed between WT and RNAi plants at each timepoint during senescence. Our findings were consistent with previous analyses of *NAM* RNAi and *NAM* mutant lines, including identifying changes to photosynthetic genes (Cantu *et al.* 2011; Pearce *et al.* 2014). However, specialized analysis techniques for time courses allow information to be shared between timepoints, which allows a more accurate and powerful analysis for datasets with larger numbers of timepoints. To take advantage of this we analyzed transcriptional changes across our 7 time points from 3 to 26 DAA in each genotype.

We found that although 52,395 (WT) and 52,626 (RNAi) genes were expressed in senescing flag leaves, only 6,508 (WT) and 2,605 (RNAi) genes were differentially expressed during this time period. In both genotypes, more genes were upregulated than downregulated, which shows that senescence is an actively regulated developmental process, as has been previously reported for wheat and other plant species (Breeze *et al.* 2011; Zhang *et al.* 2018; Borrill *et al.* 2019). Most of the genes differentially expressed in WT plants were not differentially expressed in *NAM* RNAi plants (5,140/6,508), suggesting that *NAM* genes control approximately three-quarters of the transcription response during these early stages of senescence. We observed that WT and RNAi DEGs were split into 2 waves of transcriptional changes with an initial wave of downregulation followed by upregulation during later timepoints, which might not have been evident from a less time-resolved data set. *NAM* RNAi plants maintain these transcriptional waves during senescence, albeit to a lesser extent than WT, which indicates that some transcriptional changes during senescence are *NAM*-independent, as previously proposed by Pearce *et al.* (2014). Nevertheless, the *NAM*-independent DEGs are much lower in number than DEGs in the WT time course, confirming that *NAM* genes play a major role in the transcriptional regulation of early senescence in wheat (Cantu *et al.* 2011; Pearce *et al.* 2014; Harrington, Backhaus, Singh, *et al.* 2020).

DEGs in WT were more strongly enriched for GO terms associated with hormones, nitrogen metabolism and other nutrient metabolism than DEGs in RNAi (Fig. 4). Overall genes enriched with GO terms relating to nitrogen metabolism and nutrition showed up- and downregulation in WT but most of these genes were not differentially expressed in *NAM* RNAi. This is consistent with analysis at 12 days after anthesis which identified that genes annotated to be involved in protein metabolism and catalytic process were mostly upregulated at 12 DAA in WT compared to *NAM* RNAi wheat (Cantu *et al.* 2011).

### Effect of *NAM* genes on nitrogen remobilization

Previous studies have shown that *NAM* genes affect grain protein content by altering nitrogen remobilization in a range of genetic backgrounds and environmental conditions (Uauy *et al.* 2006; Waters *et al.* 2009; Avni *et al.* 2014; Pearce *et al.* 2014; Alhabbar *et al.* 2018); yet, how this is mediated at the gene expression level is less well understood. To address this, we identified nitrogen metabolism associated genes in the RefSeqv1.1 gene annotation. In total, we identified 1,027 genes which may be involved in nitrogen transport, assimilation remobilization or transcriptional regulation in wheat by orthology to *Arabidopsis*. Approximately half of these genes were expressed in our flag leaf time course in each genotype. Over 3 times more nitrogen-associated genes were differentially expressed in WT than in RNAi across the time course (136 vs 41 genes, respectively) indicating that reduced expression of *NAM* genes affects nitrogen remobilization at the transcriptional level during senescence. The differences in nitrogen-associated gene expression between WT and RNAi may be due to direct or downstream effects of *NAM* genes which could be tested in the future using ChIP-seq or DAP-seq approaches.

We found that *NAM* genes play a significant role in controlling the expression pattern of genes associated with nitrogen transport during senescence in wheat. For example orthologs of *AAP8* (*TraesCS7B02G271151* and *TraesCS7D02G366000*) were upregulated from 10 and 13 DAA in WT, but not in RNAi. These genes had been previously shown to be highly expressed during later stages of grain development (28–30 days post-anthesis; *TaAAP21*), but their potential role in the flag leaf was not noted because flag leaf samples examined were from earlier developmental stages (Wan *et al.* 2017; Wan *et al.* 2021). Manipulating these amino acid transporters has the potential to improve grain yields, nitrogen use efficiency, and protein content in crops (Dellero 2020), and those which are *NAM* regulated (i.e. upregulated in WT but not RNAi) represent a good starting point for precise functional studies. Overall, many nitrogen transport genes were upregulated in WT but were not differentially expressed in the RNAi lines, which may indicate a true absence of transcriptional responsiveness in the RNAi line or alternatively these responses may be delayed in the RNAi line. Our analysis indicates that the widespread changes to gene expression in RNAi compared to WT are not merely a delay in timing of changes but instead represent a loss of many transcriptional responses.

Other nitrogen-associated genes showed similar trends to the transporters, with more genes differentially expressed in WT than in RNAi. For example the B homoeolog of core nitrogen assimilation gene *glutamine synthetase 1* (*GS1*) (*TraesCS6B02G327500*) was upregulated in WT but not RNAi, however the A homeolog (*TraesCS6A02G298100*) was upregulated in both WT and RNAi but to a higher maximum level in WT than RNAi. The upregulation of the A homoeolog in the RNAi as well as the WT, is consistent with *NAM* RNAi lines still being able to remobilize some nitrogen, albeit to a lower degree than WT (Waters *et al.* 2009) and with previous reports of the A homoeolog being more highly expressed than other homoeologs (Wei *et al.* 2021). We found that *glutamine synthetase 2* (*GS2*) was downregulated during senescence in WT, consistent with a previous study under high and low nitrogen (Wei *et al.* 2021). However, *GS2* was not differentially expressed in RNAi, which might indicate a loss of transcriptional control in the RNAi line across the nitrogen assimilation pathway, or a compensatory mechanism to increase nitrogen cycling.

We identified putative wheat orthologs of *Arabidopsis* transcription factors which are associated with nitrogen remobilization. However, for this set of genes the differences between WT and RNAi at the gene expression level were weaker than for nitrogen transporters or assimilation genes, suggesting either that transcription factors controlling the nitrogen pathway are less affected by *NAM* genes, or that transcription factors regulating this process are not conserved between *Arabidopsis* and wheat. We previously found that NAC transcription factors which control senescence in *Arabidopsis* are not well conserved at the expression level in wheat during senescence (Borrill *et al.* 2019); therefore, it seems likely that regulatory genes are also poorly conserved in nitrogen remobilization. Combining the differentially expressed transcription factors identified in this study with transcription factors which respond to different levels of nitrogen application (Effah *et al.* 2022) may provide a fruitful avenue to prioritize candidate genes for functional characterization.

### Conclusions

The use of time-aware differential expression analysis allows detailed analysis of the dynamics of gene expression during a developmental process such as monocarpic senescence. Here, we found that WT plants undergo stronger transcriptional changes immediately after anthesis, than *NAM* RNAi lines with delayed senescence, including genes associated with nitrogen metabolism. Nevertheless, *NAM* RNAi lines do show some gene expression changes which are associated with senescence, indicating that either the reduced levels of *NAM* gene expression in the RNAi lines are sufficient to promote senescence or that there are *NAM*-independent pathways which regulate senescence in wheat. The list of putative *NAM*-regulated genes generated in this study provides a valuable entry point to dissect the pathways regulating senescence and nutrient translocation in wheat.

## Supplementary Material

jkac275_Supplementary_Table_S1

jkac275_Supplementary_Table_S2

jkac275_Supplementary_Table_S3

jkac275_Supplementary_Table_S4

jkac275_Supplementary_Table_S5

jkac275_Supplementary_Table_S6

jkac275_Supplementary_Table_S7

jkac275_Supplementary_Table_S8

jkac275_Supplementary_Table_S9

jkac275_Supplementary_Table_S10

jkac275_Supplementary_Table_S11

jkac275_Supplementary_Table_S12

## Data Availability

RNA-seq data for RNAi samples have been deposited in the European Nucleotide Archive under project PRJEB53533. WT RNA-seq data were previously published in Borrill *et al.* (2019) and are available through PRJNA497810 in the European Nucleotide Archive. All other data are available within the article, supplemental files, and from https://doi.org/10.6084/m9.figshare.20210774.v1. All scripts are available from https://github.com/Borrill-Lab/NAM_RNAi_Senescence. Supplemental material is available at G3 online.
